# Improving dietary fiber intake is associated with a declining burden of early-onset colorectal cancer: a three-decade comparative analysis in China and globally

**DOI:** 10.1007/s00384-025-05076-5

**Published:** 2026-01-14

**Authors:** Guanmin Cui, Kai Jing, Yunxia Li, Jianhua Gu, Fang Li

**Affiliations:** 1 Department of Anorectal Surgery, Huaiyin People’s Hospital of Jinan City, No. 589, Jingsi Road, Huaiyin District, Jinan, Shandong Province China; 2 Department of Emergency Medicine, Qilu Hospital of Shandong University, 107 Wenhua West Road, Lixia District, Jinan, Shandong Province China

**Keywords:** Dietary fiber, Early-onset colorectal cancer, Global Burden of Disease, Trend analysis, China

## Abstract

**Background:**

The global incidence of early-onset colorectal cancer (EOCRC) is rising. We compared trends in dietary fiber intake, a key modifiable risk factor, and its attributable EOCRC burden among young adults in China versus globally from 1990 to 2021.

**Methods:**

Using data from the GDD and GBD 2021 for adults aged 25–49, we analyzed age-standardized mean fiber intake, summary exposure value (SEV) for low intake, and attributable EOCRC mortality and disability-adjusted life years (DALYs). Trends were quantified using the estimated annual percentage change (EAPC).

**Results:**

Between 1990 and 2018, China’s mean dietary fiber intake increased dramatically from 5.0 to 23.3 g/day (EAPC 5.73%), substantially outpacing the global increase (EAPC 1.41%). This led to a profound reversal in risk exposure; China’s SEV for low fiber declined at nearly twice the global rate (EAPC −2.14% vs −1.15%), falling below the global benchmark after 2005. Consequently, the attributable age-standardized mortality rate in China dropped from 0.15 per 100,000 to converge with the global level of 0.05 by 2021 (EAPC −3.81% vs −2.17% globally). Similar rapid declines occurred for DALYs and were more pronounced in women. Favorable epidemiological changes were the primary driver of this reduction.

**Conclusion:**

China’s success in reducing its EOCRC burden from low dietary fiber highlights nutritional improvement as a potent primary prevention strategy, reinforcing the urgent need to promote fiber-rich diets globally.

**Supplementary information:**

The online version contains supplementary material available at 10.1007/s00384-025-05076-5.

## Introduction

Colorectal cancer (CRC) remains a major global health challenge, ranking as the third most common cause of cancer-related death worldwide [1, 2]. While traditionally considered a disease of older adults, a disturbing epidemiological shift has emerged: the incidence of early-onset CRC (EOCRC), diagnosed in individuals younger than 50 years, has been alarmingly increasing across the globe over the past three decades [3, 4]. This rise is particularly pronounced in many high-income countries, but upward trends are also being reported in economically transitioning nations [5]. EOCRC often presents with distinct clinical and molecular features, including a higher likelihood of rectal location and metastatic disease at diagnosis, underscoring its clinical significance and the urgent need to understand its drivers [6].

While genetic predisposition plays a role, the rapid increase in EOCRC incidence strongly points towards the influence of modifiable environmental factors, particularly changes in lifestyle and diet experienced by recent birth cohorts [7]. Unhealthy dietary patterns are well-established risk factors for CRC, and low dietary fiber intake is a key modifiable component [8]. Dietary fiber, primarily derived from plant-based foods, is crucial for maintaining gut health and has been consistently associated with a reduced risk of CRC [9, 10]. Global nutritional guidelines universally recommend an adequate intake of dietary fiber, typically 25–35 g/day for adults, yet a substantial portion of the global population fails to meet this target [11, 12]. The potential link between widespread insufficient fiber intake and the rising EOCRC epidemic warrants systematic investigation.

China, with its vast population and unprecedented socioeconomic and nutritional transition over the past several decades, presents a unique and critical setting to examine the interplay between dietary changes and disease burden [13]. The country has experienced dramatic shifts in dietary patterns, moving away from traditional, high-fiber diets towards more processed foods, a phenomenon mirrored in many developing nations. Comparing the long-term trends of dietary fiber intake and the attributable burden of EOCRC in China with the overall global patterns can provide invaluable insights. Such a comparison can help elucidate how public health policies and population-level dietary shifts in a major transitioning economy impact the trajectory of a rising cancer, offering lessons that may be highly relevant for other countries undergoing similar transformations.

To date, while some studies have described the overall burden of EOCRC or the diet-related CRC burden for all ages [2, 14], a specific, long-term analysis of the EOCRC burden attributable to low dietary fiber, particularly with a direct comparison between China and the global landscape, is lacking. A detailed understanding of these trends is essential for developing targeted, evidence-based public health strategies. Therefore, this study aimed to specifically address the burden of EOCRC by focusing on the standard age definition of this condition (< 50 years). Using data from the Global Dietary Database (GDD) and the Global Burden of Disease (GBD), we systematically analyzed trends in dietary fiber intake and its attributable burden in adults aged 25–49 years—the primary age group for which GBD provides risk-attributable estimates for EOCRC in China and globally from 1990 to 2021.

## Methods

### Study overview and data sources

This study utilized publicly available data from the GDD 2018 and the GBD 2021. The GBD is a comprehensive, systematic effort coordinated by the Institute for Health Metrics and Evaluation (IHME) that quantifies health loss from hundreds of diseases, injuries, and risk factors across 204 countries and territories, providing comparable estimates of mortality, morbidity (e.g., disability-adjusted life years (DALYs)), and risk-attributable burden by age, sex, and year. The GDD is a standardized global repository that compiles and harmonizes individual-level dietary intake data from national surveys, offering comparable estimates of consumption for key foods and nutrients—including dietary fiber—across populations and time. We conducted a comparative analysis of dietary fiber intake and its associated burden of EOCRC in China and globally from 1990 to 2021. Data on dietary fiber consumption for adults aged 25–49 years were extracted from the GDD, covering the period 1990–2018 across 185 countries. Dietary fiber was defined according to the GDD methodology, primarily based on national nutrition surveys and food availability data.

Estimates of EOCRC mortality and disability-adjusted life years (DALYs) attributable to low dietary fiber intake were obtained from the GBD 2021 study via the Global Health Data Exchange (GHDx) platform. These data spanned 1990–2021 for 204 countries and territories. It is a standard GBD methodology that risk factor data from the most recent comprehensive source (i.e., GDD 2018) are used to model the attributable burden for the entire study period, including the most recent years (2019–2021), ensuring methodological consistency across the time series. The analysis was strictly limited to the 25–49 age group for two critical reasons: (1) this aligns with the universally accepted clinical definition of early-onset colorectal cancer (diagnoses under age 50), which is the explicit focus of our investigation; and (2) it corresponds to the specific age stratification for which the GBD provides risk-attributable burden estimates for this disease, making it the most appropriate and scientifically rigorous range for this analysis. Socio-demographic Index (SDI) data for 2021 were also retrieved from the GBD to stratify countries into five quintiles.

### Assessment of dietary fiber intake and risk exposure

We analyzed temporal trends in mean dietary fiber intake (grams/day), age-standardized to the GBD 2021 reference population. To quantify the health risks associated with suboptimal intake, we evaluated the summary exposure value (SEV). The SEV is a metric scaled from 0 (no risk) to 100 (maximum risk) that quantifies the population-level risk exposure, considering the distribution of intake levels and the dose-response relationship between fiber intake and disease risk. Age-standardized SEVs were analyzed to assess trends in risk exposure over time.

### Analysis of attributable disease burden

We extracted age-standardized mortality rates (ASMR) and DALY rates (per 100,000 population) for EOCRC attributable to low dietary fiber. The GBD comparative risk assessment framework was used to estimate the proportion of disease burden caused by this dietary risk factor.

### Statistical analysis

To quantify temporal trends for intake, SEV, and disease burden rates, we calculated the estimated annual percentage change (EAPC). The EAPC was derived from a linear regression model fitted to the natural logarithm of the rates against the calendar year, using the formula: EAPC = 100 × (exp(*β*) − 1), where *β* is the regression coefficient. A non-zero EAPC with a 95% confidence interval (CI) that did not include 0 was considered to indicate a significant trend.

A demographic decomposition analysis was performed to disentangle the contributions of three key drivers to the change in absolute attributable EOCRC deaths and DALYs between 1990 and 2021: population growth, population aging, and changes in age-specific epidemiological rates [15].

Furthermore, a Bayesian age-period-cohort (BAPC) model was employed to project the burden of attributable EOCRC through 2035 [16]. This hierarchical model concurrently captured the non-linear effects of age, calendar period, and birth cohort to generate robust forecasts. Projections were accompanied by 95% uncertainty intervals (UIs), derived from the posterior predictive distributions.

All estimates from the GDD and GBD are presented with their 95% UIs, which reflect the cumulative uncertainty from input data, corrections for bias, and modeling parameters. All statistical analyses were conducted using R software (version 4.2.1) and JD_GBDR (v2.38, Jingding Medical Technology, China). This study adheres to the Guidelines for Accurate and Transparent Health Estimates Reporting (GATHER) and the Strengthening the Reporting of Observational Studies in Epidemiology (STROBE) guidelines.

### Ethical considerations

This study was based on publicly available, de-identified aggregate data. Therefore, institutional review board approval was not required.

## Results

### Dietary fiber intake in China and globally, 1990–2018

**Fig. 1:**
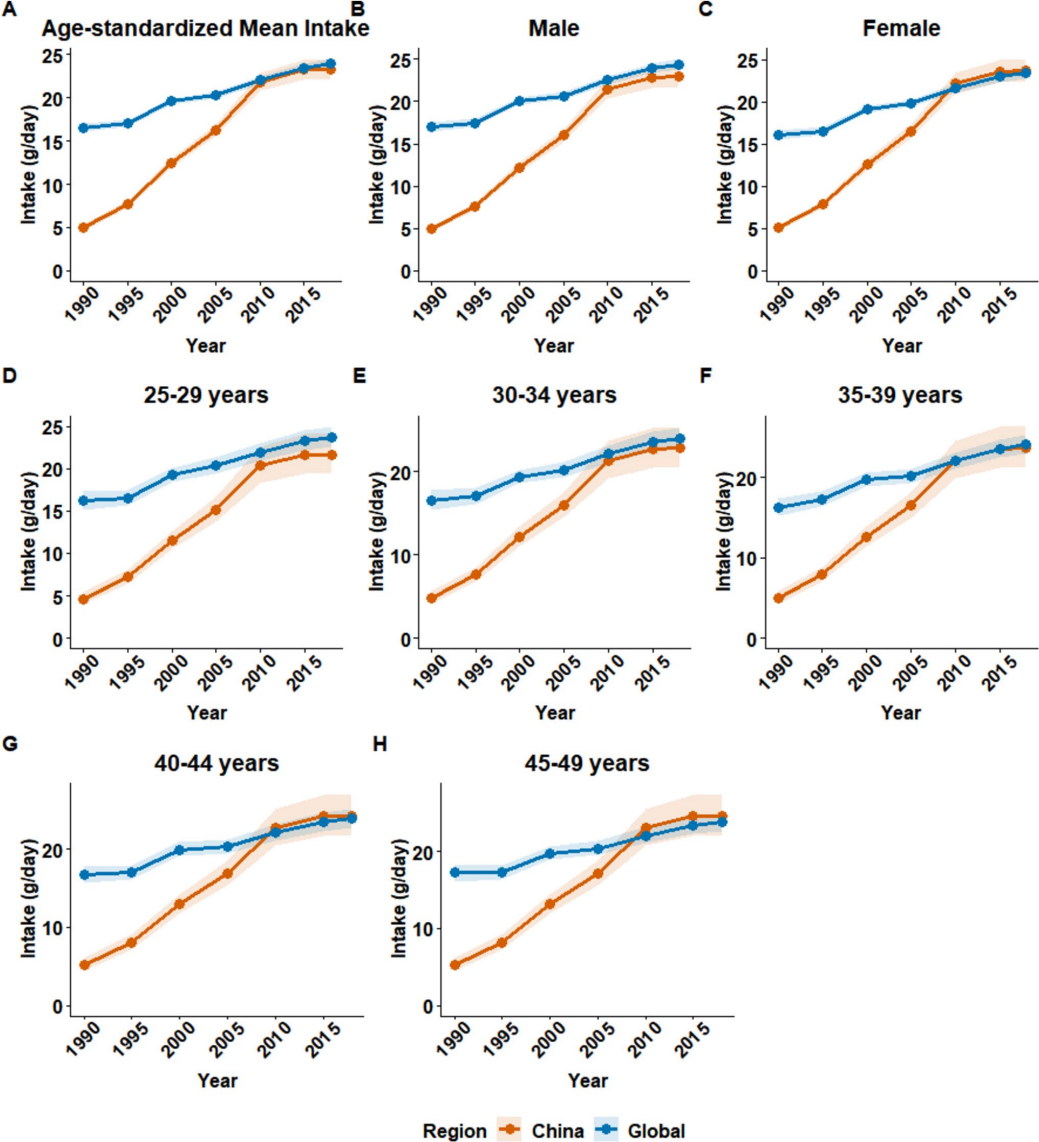
Temporal trends in mean dietary fiber intake among adults aged 25–49 years in China and globally, 1990–2018. The figure displays the trends in mean daily dietary fiber intake (grams/day). **A** The age-standardized mean intake for the total population in China versus globally. **B**, **C** Age-standardized trends stratified by sex for men and women, respectively. **D**–**H** The unstandardized mean intake for specific 5-year age groups: 25–29, 30–34, 35–39, 40–44, and 45–49 years. Shaded areas represent 95% uncertainty intervals. Data were sourced from the Global Dietary Database (GDD) 2018

Between 1990 and 2018, the mean dietary fiber intake among adults aged 25–49 years increased substantially in China and globally (Table 1). In China, the age-standardized mean intake experienced a remarkable increase, rising from a very low baseline of 5.0 g/day (95% CI 4.7–5.3) in 1990 to 23.3 g/day (22.2–24.4) in 2018, with an estimated annual percentage change (EAPC) of 5.73 (95% CI 3.81–7.68). Globally, the increase was more modest, from 16.6 g/day (16.0–17.1) in 1990 to 23.9 g/day (23.4–24.4) in 2018 (EAPC 1.41, 1.15–1.66) (Fig. 1A). Consequently, China’s intake, which was considerably lower than the global average before 2010, approached the global level by 2018.

**Table 1:** Temporal trends in dietary fiber intake among adults aged 25–49 years in China and globally, 1990–2018

	China			Global		
	1990	2018	EAPC (95% CI)	1990	2018	EAPC (95% CI)
Total	5.0 (4.7, 5.3)	23.3 (22.2, 24.4)	5.73 (3.81, 7.68)	16.6 (16.0, 17.1)	23.9 (23.4, 24.4)	1.41 (1.15, 1.66)
Sex						
Male	4.9 (4.5, 5.3)	22.9 (21.6, 24.2)	5.73 (3.81, 7.67)	17.0 (16.4, 17.5)	24.4 (23.8, 25.0)	1.38 (1.13, 1.63)
Female	5.1 (4.7, 5.5)	23.7 (22.3, 25.1)	5.73 (3.82, 7.68)	16.1 (15.6, 16.6)	23.4 (22.8, 24.0)	1.43 (1.17, 1.69)
Age						
25–29 years	4.6 (4.0, 5.4)	21.7 (19.4, 24.1)	5.73 (3.81, 7.68)	16.2 (15.1, 17.4)	23.7 (22.5, 25.0)	1.48 (1.19, 1.78)
30–34 years	4.9 (4.2, 5.7)	22.8 (20.4, 25.4)	5.73 (3.81, 7.68)	16.6 (15.4, 17.8)	24.0 (22.8, 25.3)	1.43 (1.21, 1.65)
35–39 years	5.1 (4.3, 5.9)	23.6 (21.2, 26.3)	5.73 (3.81, 7.68)	16.3 (15.2, 17.4)	24.1 (22.9, 25.3)	1.44 (1.2, 1.68)
40–44 years	5.2 (4.4, 6.0)	24.2 (21.7, 27.0)	5.73 (3.81, 7.68)	16.7 (15.6, 17.8)	23.9 (22.8, 25.1)	1.38 (1.07, 1.69)
45–49 years	5.3 (4.5, 6.1)	24.6 (22.1, 27.5)	5.73 (3.82, 7.68)	17.2 (16.2, 18.4)	23.8 (22.6, 25.0)	1.27 (1.01, 1.54)

Data presented as mean daily intake (95% CI) in grams per day (g/d)

EAPC calculated using linear regression models on log-transformed intake values. Data represent population-weighted estimates for adults aged 25–49 years

*EAPC* estimated annual percentage change, *CI* confidence interval

This trend was consistent across both sexes (Table 1). By 2018, the mean intake in Chinese women (23.7 g/day, 22.3–25.1) slightly surpassed the global average for women (23.4 g/day, 22.8–24.0), whereas intake in Chinese men (22.9 g/day, 21.6–24.2) remained slightly below their global counterparts (24.4 g/day, 23.8–25.0) (Fig. 1B, C). Analysis by age group revealed that while all groups in China showed a steep increase in fiber consumption, the gap with global levels narrowed more rapidly in older age brackets. For instance, in the 45–49 years age group, China’s mean intake rose from 5.3 g/day (4.5–6.1) to 24.6 g/day (22.1–27.5), slightly exceeding the global figure of 23.8 g/day (22.6–25.0) by 2018 (Table 1, Fig. 1D–H). Despite these improvements, the mean dietary fiber intake in both China and globally remained below the recommended daily amount of 25–30 g for adults. In a global context, China’s dietary fiber intake in 2018 was at a moderate level, higher than that in most of East Asia and North America but lower than in regions such as South Asia and parts of Africa (Supplementary Fig. 1).

### Reversal of risk exposure, 1990–2021

**Fig. 2:**
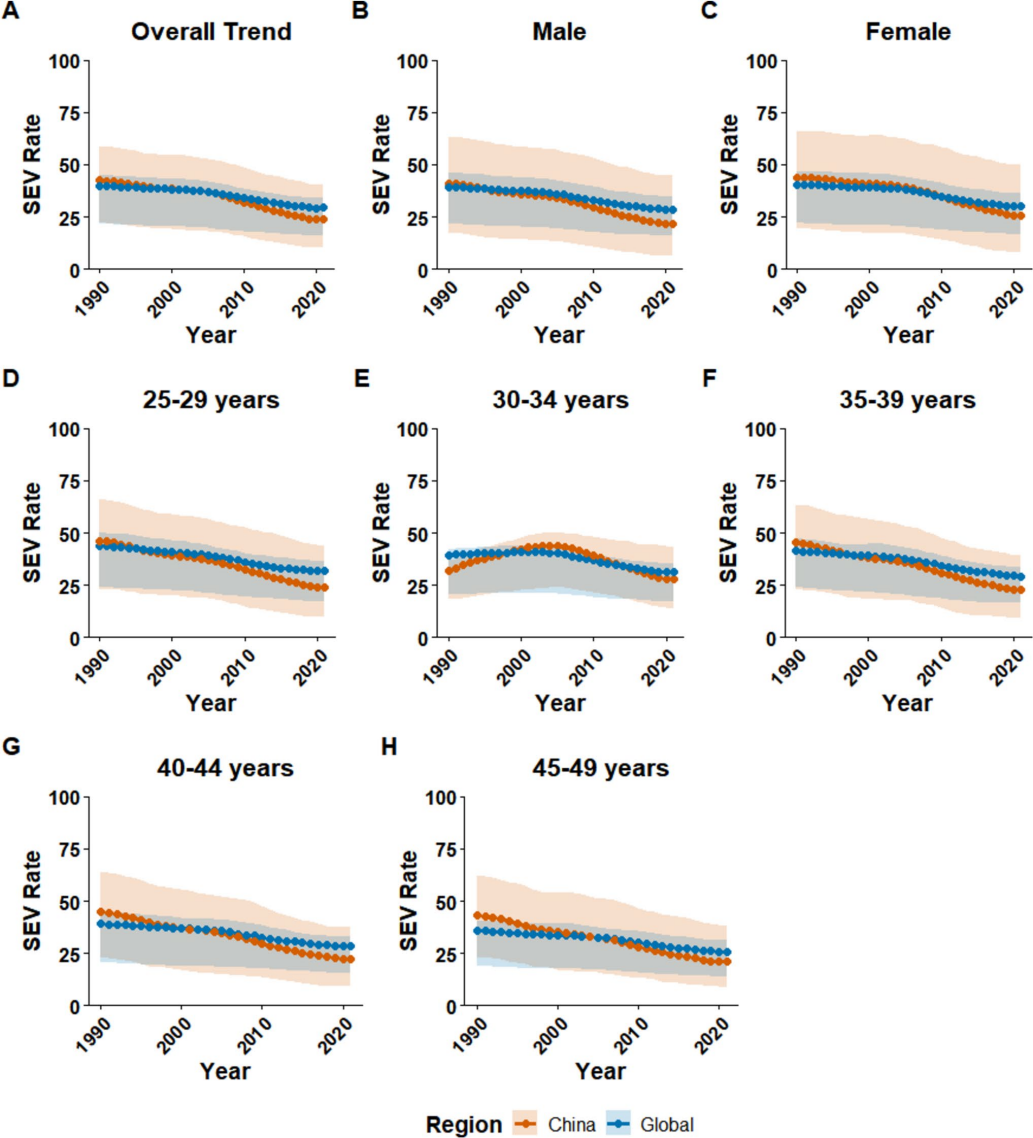
Temporal trends in summary exposure value (SEV) for low dietary fiber intake among adults aged 25–49 years in China and globally, 1990–2021. The figure illustrates the trends in the summary exposure value (SEV), a measure of population risk exposure scaled from 0 (no risk) to 100 (maximum risk). **A** The age-standardized SEV for the total population in China versus globally. **B**, **C** Age-standardized trends stratified by sex. **D**–**H** The unstandardised SEV for specific 5-year age groups: 25–29, 30–34, 35–39, 40–44, and 45–49 years. Shaded areas represent 95% uncertainty intervals. SEV data were sourced from the Global Burden of Disease (GBD) Study 2021

Consistent with the observed trends in dietary intake, the summary exposure value (SEV) for low dietary fiber intake in China for adults aged 25–49 years showed a marked decline between 1990 and 2021. The age-standardized SEV decreased from 46.3 (95% CI 23.0–66.0) in 1990 to 24.0 (10.2–43.7) in 2021, with an EAPC of −2.14% (95% CI −2.27 to −2.01) (Table 2, Fig. 2A). This rate of decline was nearly double the global rate, which saw the SEV decrease from 43.8 (23.9–49.8) to 31.8 (17.2–36.5) over the same period (EAPC −1.15%, −1.22 to −1.08).

**Table 2:** Temporal trends in summary exposure values for dietary fiber intake among adults aged 25–49 in China and globally, 1990–2021

	China			Global		
	1990	2018	EAPC (95% CI)	1990	2018	EAPC (95% CI)
Total	46.3 (23.0, 66.0)	24.0 (10.2, 43.7)	−2.14% (−2.27%, −2.01%)	43.8 (23.9, 49.8)	31.8 (17.2, 36.5)	−1.15% (−1.22%, −1.08%)
Sex						
Male	44.3 (17.8, 71.4)	21.8 (6.3, 45.9)	−2.33% (−2.45%, −2.21%)	43.1 (23.5, 50.9)	31.3 (17.8, 37.7)	−1.15% (−1.22%, −1.09%)
Female	48.4 (19.8, 75.4)	26.5 (8.1, 54.3)	−1.97% (−2.11%, −1.84%)	44.4 (25.2, 52.1)	32.3 (18.2, 38.1)	−1.15% (−1.23%, −1.07%)
Age						
25–29 years	46.3 (23.0, 66.0)	24.0 (10.2, 43.7)	−2.14% (−2.27%, −2.01%)	43.8 (23.9, 49.8)	31.8 (17.2, 36.5)	−1.15% (−1.22%, −1.08%)
30–34 years	31.9 (18.0, 36.7)	27.8 (13.9, 43.2)	−0.70% (−1.18%, −0.22%)	39.3 (20.7, 42.1)	31.2 (17.1, 35.6)	−0.90% (−1.07%, −0.72%)
35–39 years	45.2 (22.7, 63.1)	23.0 (9.5, 39.2)	−2.26% (−2.39%, −2.13%)	41.3 (23.9, 47.1)	29.3 (16.4, 33.7)	−1.19% (−1.28%, −1.10%)
40–44 years	44.9 (23.2, 63.8)	22.5 (9.5, 37.6)	−2.33% (−2.44%, −2.23%)	39.1 (20.8, 44.5)	28.3 (15.3, 32.9)	−1.13% (−1.22%, −1.05%)
45–49 years	43.3 (23.3, 62.5)	21.1 (9.0, 38.2)	−2.41% (−2.51%, −2.31%)	35.9 (18.9, 40.6)	25.9 (13.9, 31.3)	−1.12% (−1.21%, −1.03%)

Data presented as age-standardized exposure levels on a scale from 0 (minimal exposure) to 100 (maximum exposure)

*EAPC* estimated annual percentage change, *CI* confidence interval

This rapid improvement led to a reversal in risk exposure levels. While China’s SEV was higher than the global average in 1990, it fell below the global benchmark around 2005 and remained lower thereafter (Fig. 2A). By sex, the improvement was more rapid among women in China, whose SEV dropped below the global female average as early as the mid-1990s. In contrast, the SEV for Chinese men fell below the global male average more recently, around 2010 (Fig. 2B, C).

The declining trend in SEV was observed across most age groups in China (Table 2). A distinct pattern was noted in the 30–34 age group, where the SEV initially increased from 1990 to the early 2000 s before following the overall downward trend, falling below the global level after 2015 (Fig. 2F). For all other age groups, the SEV in China was consistently higher than the global average in the early years of the study period before declining to lower levels in the later years (Fig. 2D, E, G, H).

### Declining burden of attributable early-onset colorectal cancer, 1990–2021

**Fig. 3:**
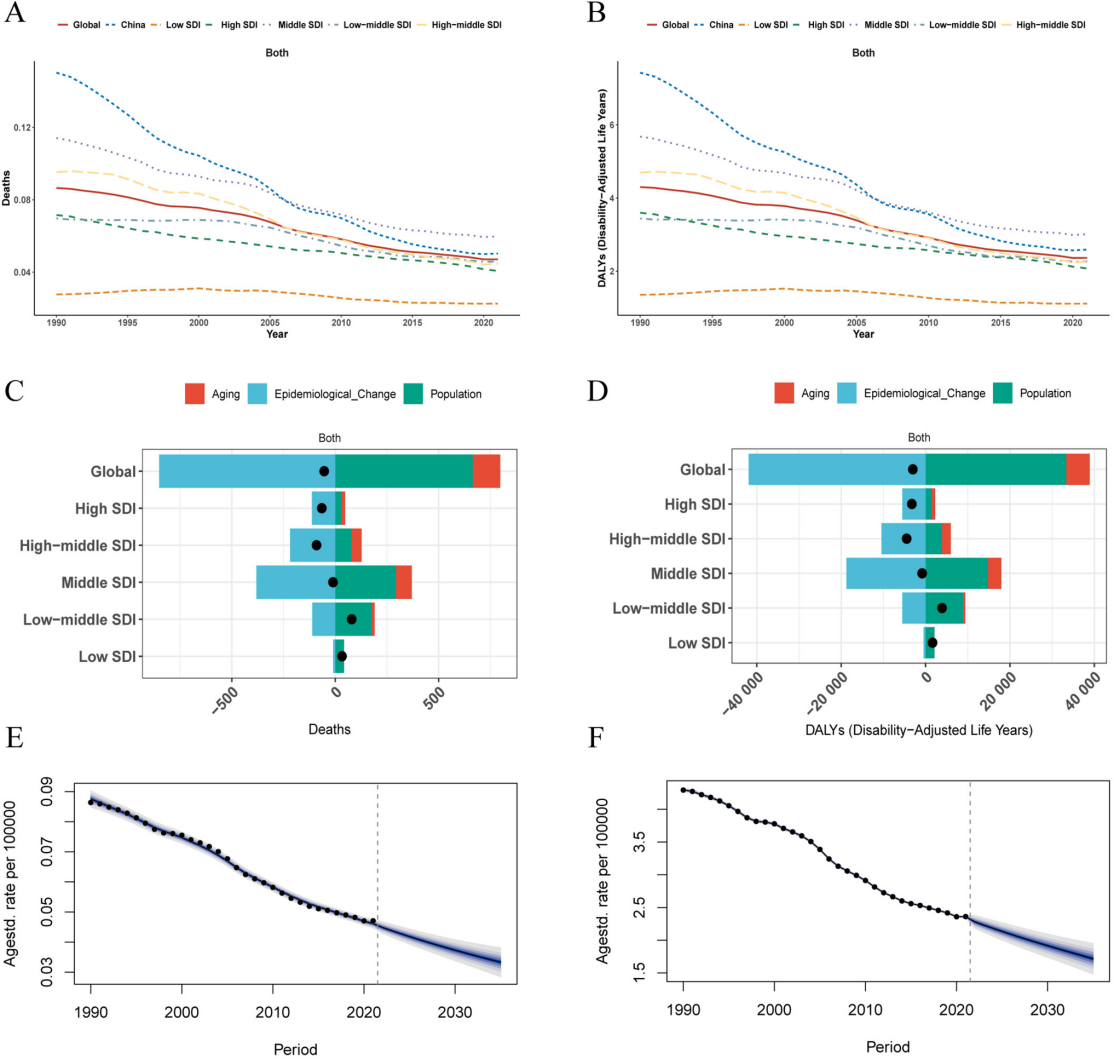
Disease burden of early-onset colorectal cancer attributable to low dietary fiber, 1990–2021, and projections to 2035. **A**, **B** The temporal trends in age-standardized mortality rates (ASMR) and DALY rates, respectively, for China, globally, and by Socio-demographic Index (SDI) quintiles. **C**, **D** The decomposition analysis, attributing the change in absolute numbers of attributable deaths and DALYs between 1990 and 2021 to population growth, population aging, and epidemiological changes. **E**, **F** The projected trends of attributable ASMR and DALY rates through 2035 based on a Bayesian age-period-cohort model. Shaded areas represent 95% uncertainty intervals. DALYs, disability-adjusted life years. Data were sourced from the Global Burden of Disease (GBD) Study 2021

In 1990, the age-standardized mortality rate (ASMR) of early-onset colorectal cancer (EOCRC) attributable to low dietary fiber in China (0.15 per 100,000; 95% CI 0.05–0.28) was substantially higher than the global average (0.09 per 100,000; 0.04–0.14) (Table 3). However, China experienced a much steeper decline over the subsequent three decades, with an EAPC of −3.81% (95% CI −3.93 to −3.70), nearly twice the global rate of decline (EAPC −2.17%, −2.27 to −2.07). By 2021, China’s ASMR had converged with the global level (0.05 per 100,000) (Fig. 3A). A similar pattern was observed for disability-adjusted life years (DALYs), where China’s age-standardized rate dropped from 7.42 per 100,000 in 1990 to 2.59 per 100,000 in 2021, with an EAPC of −3.70% (vs global EAPC of −2.15%) (Table 4, Fig. 3B).

**Table 3:** Mortality trends of early-onset colorectal cancer attributable to low dietary fiber intake in China and globally, 1990–2021

	China			Global		
	1990	2018	EAPC (95% CI)	1990	2018	EAPC (95% CI)
Overall	0.15 (0.05, 0.28)	0.05 (0.02, 0.12)	−3.81 (−3.93, −3.70)	0.09 (0.04, 0.14)	0.05 (0.02, 0.08)	−2.17 (−2.27, −2.07)
Sex						
Male	0.17 (0.05, 0.36)	0.07 (0.01, 0.19)	−3.10 (−3.22, −2.99)	0.09 (0.04, 0.16)	0.05 (0.02, 0.09)	−1.86 (−1.95, −1.78)
Female	0.13 (0.04, 0.27)	0.03 (0.01, 0.09)	−4.98 (−5.13, −4.82)	0.08 (0.04, 0.14)	0.04 (0.02, 0.06)	−2.54 (−2.66, −2.41)
Age group						
25–29 years	0.04 (0.01, 0.08)	0.01 (0.00, 0.03)	−3.63 (−3.83, −3.42)	0.02 (0.01, 0.04)	0.01 (0.01, 0.02)	−2.52 (−2.64, −2.40)
30–34 years	0.05 (0.02, 0.08)	0.03 (0.01, 0.07)	−1.86 (−2.35, −1.36)	0.04 (0.02, 0.06)	0.02 (0.01, 0.04)	−1.81 (−2.14, −1.48)
35–39 years	0.15 (0.05, 0.29)	0.05 (0.01, 0.11)	−4.16 (−4.39, −3.94)	0.08 (0.04, 0.13)	0.04 (0.02, 0.07)	−2.48 (−2.63, −2.34)
40–44 years	0.24 (0.08, 0.43)	0.07 (0.02, 0.18)	−4.18 (−4.39, −3.96)	0.13 (0.06, 0.20)	0.07 (0.03, 0.11)	−2.26 (−2.34, −2.18)
45–49 years	0.34 (0.11, 0.64)	0.10 (0.03, 0.25)	−4.01 (−4.18, −3.84)	0.20 (0.09, 0.32)	0.11 (0.05, 0.17)	−2.00 (−2.07, −1.93)

Data presented as age-standardized mortality rates per 100,000 person-years (95% CI)

*EAPC* estimated annual percentage change, *CI* confidence interval

**Table 4:** Disease burden trends of early-onset colorectal cancer attributable to low dietary fiber intake in China and globally, 1990–2021

	China			Global		
	1990	2018	EAPC (95% CI)	1990	2018	EAPC (95% CI)
Overall	7.42 (2.52, 13.81)	2.59 (0.78, 6.11)	−3.70 (−3.82, −3.58)	4.30 (1.88, 6.89)	2.36 (1.02, 3.81)	−2.15 (−2.25, −2.04)
Sex						
Male	8.29 (2.24, 17.86)	3.49 (0.75, 9.63)	−2.98 (−3.10, −2.86)	4.52 (1.94, 7.77)	2.69 (1.16, 4.68)	−1.84 (−1.93, −1.75)
Female	6.48 (1.77, 13.51)	1.64 (0.34, 4.39)	−4.86 (−5.01, −4.70)	4.07 (1.75, 6.73)	2.03 (0.88, 3.22)	−2.52 (−2.65, −2.39)
Age						
25–29 years	2.59 (0.81, 4.91)	0.93 (0.25, 2.21)	−3.57 (−3.77, −3.36)	1.47 (0.63, 2.41)	0.75 (0.33, 1.21)	−2.49 (−2.61, −2.37)
30–34 years	2.92 (1.24, 4.77)	2.01 (0.65, 4.25)	−1.79 (−2.28, −1.30)	2.20 (1.00, 3.37)	1.48 (0.63, 2.37)	−1.78 (−2.11, −1.46)
35–39 years	7.91 (2.57, 15.39)	2.57 (0.72, 6.09)	−4.11 (−4.33, −3.89)	4.35 (1.92, 7.20)	2.18 (0.94, 3.64)	−2.23 (−2.31, −2.15)
40–44 years	11.61 (3.91, 20.94)	3.65 (1.02, 8.71)	−4.11 (−4.33, −3.89)	6.26 (2.70, 9.84)	3.34 (1.48, 5.39)	−2.23 (−2.31, −2.15)
45–49 years	14.80 (4.99, 28.18)	4.50 (1.48, 11.10)	−3.93 (−4.11, −3.76)	8.72 (3.80, 14.06)	4.90 (2.09, 7.75)	−1.96 (−2.03, −1.90)

Data presented as age-standardized disability-adjusted life years (DALYs) rates per 100,000 person-years (95% CI)

*EAPC* estimated annual percentage change, *CI* confidence interval

The reduction in disease burden was more pronounced in women than in men in China. The EAPC for attributable mortality was −4.98% for women versus −3.10% for men, and for DALYs, −4.86% versus −2.98%, respectively (Tables 3 and 4). The attributable burden increased with age, though declining trends were evident across all age groups. Notably, the 30–34 age group showed the slowest rate of decline for both mortality and DALYs.

When benchmarked against Socio-demographic Index (SDI) regions, China’s trajectory of decline in attributable burden was steeper than that of high-SDI and middle-SDI regions, positioning it as a leader in risk reduction among developing economies (Fig. 3A, B). Spatially, the EAPC of attributable mortality and DALYs in China was among the most negative globally, indicating one of the fastest rates of reduction, particularly when compared to North America, Europe, and other parts of East Asia (Supplementary Fig. 2).

Decomposition analysis revealed that the substantial decrease in the attributable EOCRC burden in China and globally was overwhelmingly driven by favorable epidemiological changes—that is, reductions in age-specific incidence and mortality rates. This factor’s positive impact far outweighed the upward pressure from population growth and aging (Fig. 3C, D). In stark contrast, in low-SDI regions, population growth was the dominant driver of the increasing burden.

Looking forward, Bayesian age-period-cohort modeling projects a continued global decline in the attributable burden. By 2035, the global age-standardized mortality rate is predicted to fall to 0.033 per 100,000 (95% UI 0.028–0.039), and the DALY rate is projected to decrease to 1.72 per 100,000 (95% CI 1.62–1.81), suggesting sustained positive momentum (Fig. 3E, F).

## Discussion

In this comprehensive analysis of GBD and GDD data, we unveiled a remarkable public health transition in China concerning dietary fiber intake and its associated burden of EOCRC over the past three decades. Our study reveals three core findings. First, China has experienced a dramatic increase in dietary fiber consumption among young adults, rising from a critically low level in 1990 to approach the global average by 2018. Second, this nutritional improvement translated directly into a profound reversal of risk exposure, with China’s SEV for low dietary fiber declining at nearly twice the global rate and falling below the global benchmark after 2005. Finally, these positive changes were associated with a steep reduction in the attributable burden of EOCRC, where China’s initially high mortality and DALY rates declined significantly, eventually converging with global levels. This progress was overwhelmingly driven by favorable epidemiological changes rather than demographic shifts.

The dramatic increase in dietary fiber intake among young adults in China, from a critically low level in 1990 to near-global parity by 2018, represents a profound nutritional transition with significant public health implications. This trajectory is not a monolithic event but likely reflects China’s multi-stage socioeconomic evolution. The extremely low baseline in the early 1990 s corresponds to a period of more limited food security and diversity. Subsequent rapid economic growth fundamentally reshaped the national food environment, substantially increasing the availability, affordability, and variety of fiber-rich foods such as fresh fruits, vegetables, and whole grains [17, 18]. More recently, this upward trend has likely been further sustained by rising public health awareness and the implementation of national policies, including the widely promoted Chinese Dietary Guidelines [19]. This complex evolution challenges a simplistic narrative of dietary Westernization, suggesting that alongside the adoption of some unhealthy processed foods, a significant and positive shift towards improved nutritional quality has occurred [20, 21]. Nevertheless, it is crucial to temper this optimistic finding with the observation that the average intake in 2018 still fell short of the nationally recommended 25–30 g/day [17, 22]. This persistent fiber gap indicates that while substantial progress has been achieved, continued public health efforts are essential to fully leverage the preventive potential of dietary fiber [23, 24].

A key strength of our study is the clear demonstration of a complete public health success narrative, linking improved nutrition directly to reduced cancer risk and burden. The reversal in the SEV—from being higher than the global average to significantly lower—serves as a powerful quantitative testament to the real-world impact of the dietary shifts observed. This finding provides a crucial intermediate bridge between population-level dietary intake and its ultimate health outcomes. Furthermore, our decomposition analysis reinforces this conclusion by identifying favorable epidemiological changes, rather than demographic factors like population aging, as the overwhelming driver of the decline in attributable EOCRC burden. This robustly suggests that the observed reduction represents a genuine improvement in population health, likely stemming from primary prevention efforts.

While increased dietary fiber is a major plausible contributor to this positive epidemiological trend [25–27], it is important to acknowledge that other concurrent factors, such as advancements in early detection, improved access to healthcare, and enhanced treatment modalities for colorectal cancer, may have also played a synergistic role, even if their direct impact on the under-50 population is still evolving. The protective effects of dietary fiber are thought to be mediated by multiple mechanisms, including modulation of the gut microbiome [28], suppression of aberrant WNT pathway activation [29, 30], and a reduction in the risk of postoperative complications [25]. Indeed, the association between higher preoperative fiber intake and lower risk of postoperative complications in colorectal cancer patients provides emerging evidence for the importance of nutritional intervention throughout the continuum of cancer care [25]. Nonetheless, our findings provide compelling, long-term evidence that population-wide dietary modification is a potent lever for mitigating the burden of early-onset cancer. This conclusion is reinforced by multiple lines of evidence, such as the differential risk of colorectal cancer molecular subtypes associated with fiber intake [26] and the correlation between intake disparities and cancer incidence across different ethnic groups [31], both highlighting the public health value of dietary interventions. Although the protective effect in Asian populations remains a subject of debate [32], our study, by quantifying the change in SEV, contributes novel epidemiological evidence supporting the pivotal role of dietary fiber in the primary prevention of EOCRC.

The granular disparities observed across sex and age groups in our analysis provide valuable insights into the socio-behavioral dynamics underpinning dietary change. The consistently faster improvement in fiber intake and subsequent reduction in risk exposure and disease burden among women compared to men aligns with broader evidence suggesting that women often exhibit greater health consciousness and are more receptive to dietary guidance [33]. This gender gap highlights the need for targeted health promotion strategies that effectively engage men. Furthermore, the divergent trends across age strata are particularly revealing. The pronounced catch-up in the 40–49 age group may reflect a heightened perception of health risks as individuals approach middle age, motivating more proactive dietary modifications [34]. In stark contrast, the relatively sluggish progress in the 30–34 age group is concerning and may mirror the unique lifestyle pressures faced by this cohort, such as intense work schedules, reliance on convenience foods, and less structured eating patterns, which are known barriers to healthy eating [35]. These findings underscore that a one-size-fits-all public health approach may be insufficient. Future interventions should be tailored to address the specific barriers and motivators prevalent in different demographic segments [36].

Viewed through a global lens, China’s trajectory offers a compelling and cautiously optimistic blueprint for other nations undergoing rapid economic and nutritional transitions. The findings demonstrate that it is possible for a large, developing country to decouple rapid economic growth from a universally negative dietary trajectory [37, 38]. It is also instructive to compare these findings with other populations that have implemented nutritional interventions. For instance, despite long-standing public health campaigns promoting fruit and vegetable consumption in high-income nations, such as the “5 A Day” program in the USA and the UK, population-level fiber intake often remains stagnant and well below recommended targets. This stagnation coincides with the strictly rising incidence of EOCRC in these regions, contrasting sharply with the decreasing attributable burden observed in China. This disparity suggests that effective intervention requires not just guidelines, but structural shifts in food availability and accessibility, as experienced during China’s rapid economic transition. Instead of succumbing entirely to the pitfalls of an unhealthy Westernized diet, China’s experience suggests that concerted public health efforts—including national dietary guidelines, widespread health education, and a responsive food industry—can effectively steer population-level dietary patterns towards a healthier direction, even amidst profound societal change [39, 40]. This model holds particular relevance for other middle-SDI countries facing similar dual challenges of communicable and non-communicable diseases [41, 42], where dietary transitions are characterized by both reduced carbohydrate intake and concerning increases in fat consumption [37, 38]. However, this success story should not breed complacency. Our projections indicate that the global burden of EOCRC will remain a significant challenge, particularly in middle-SDI countries where mortality rates for nutrition-related diseases persist at elevated levels [43, 44]. The lessons from China underscore the need for proactive and sustained global commitment to promoting high-fiber diets, as evidenced by studies showing traditional plant-based dietary patterns are associated with reduced health risks [39], while Westernized diets high in red/processed meat disproportionately affect middle-SDI populations [45]. This dietary approach represents a cornerstone strategy in the ongoing fight against premature cancer, especially crucial for regions where socioeconomic development has paradoxically exacerbated nutritional inequalities [46, 47].

Our study has several limitations that warrant discussion. First and foremost, as an ecological study using aggregated national-level data, the findings represent population-level associations and cannot establish causality at the individual level. Consequently, we could not adjust for individual-level confounders known to influence CRC risk, such as genetic predisposition, physical activity levels, or the intake of other dietary components like red and processed meat. Moreover, the observed decline in the attributable EOCRC burden is likely multifactorial. Concurrent public health improvements, such as changes in other lifestyle factors, increased disease awareness, and advancements in early detection and treatment, may have also contributed to the observed trends, even if organized screening is less common for this age group. Second, the estimates are subject to the inherent limitations of the data sources. GBD’s attributable burden figures are derived from complex modeling with its own assumptions and uncertainties, particularly in regions with sparse primary data. Similarly, dietary intake data from the GDD are prone to measurement errors from self-reporting and variations in food composition databases. Third, while our analysis was appropriately restricted to the 25–49 age group to align with the standard definition of EOCRC, this focus may miss emerging trends in even younger cohorts (i.e., under 25), a point that warrants future investigation.

In conclusion, China has made substantial progress in improving dietary fiber intake and consequently reducing the attributable burden of EOCRC among its young adult population over the last three decades, narrowing a significant historical gap with global levels. These findings highlight the profound impact of nutritional transition on cancer epidemiology and underscore the critical importance of sustained public health efforts promoting high-fiber diets as a key strategy for the primary prevention of EOCRC, both in China and globally.

## Supplementary information

Below is the link to the electronic supplementary material.

ESM 1 (DOCX 475 KB)

## Data Availability

The datasets analyzed during the current study are publicly available. Data from the GBD 2021 study can be accessed via the GBD results tool (https://vizhub.healthdata.org/gbd-results/). Data from the Global Dietary Database can be accessed at its official website (https://www.globaldietarydatabase.org/).
