# Supplementary material for: Improving dietary fiber intake is associated with a declining burden of early-onset colorectal cancer: a three-decade comparative analysis in China and globally
Source: Int J Colorectal Dis. 2026 Jan 14;41(1):33. doi: 10.1007/s00384-025-05076-5 (PMC12808260; doi:10.1007/s00384-025-05076-5)
Supplement: Supplementary file 1 — (DOCX 475 KB) [file 384_2025_5076_MOESM1_ESM.docx]

**Supplementary Materials​​**


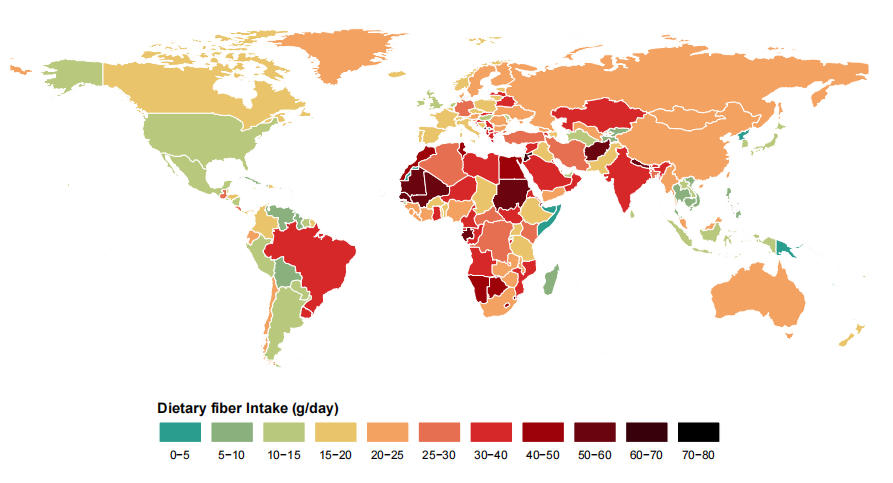


**Supplementary Figure 1.**  Global Heterogeneity in Dietary Fiber Intake Among Adults Aged 25–49 Years (2018)​


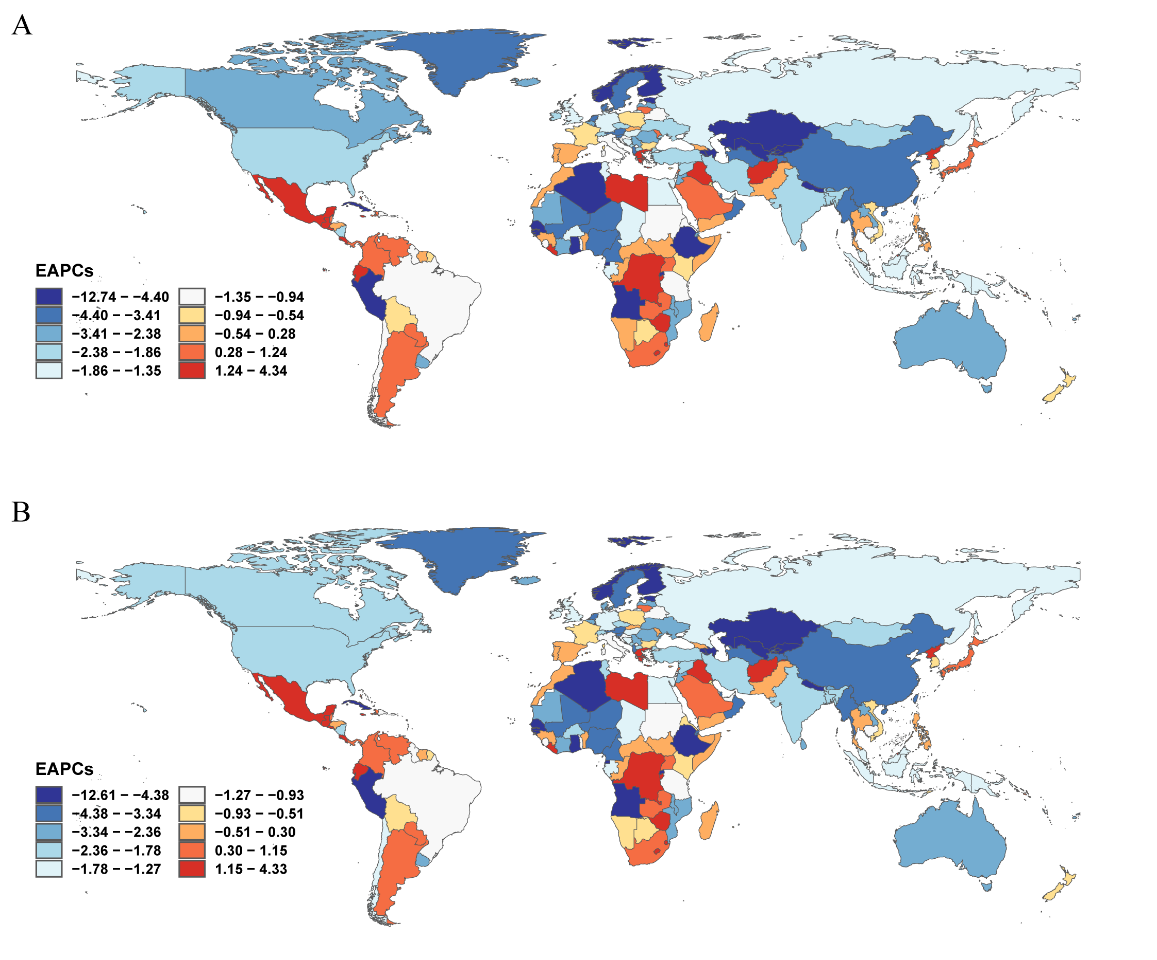


Supplementary Figure 2. Global Map of the Estimated Annual Percentage Change (EAPC) in the Burden of Early-Onset Colorectal Cancer Attributable to Low Dietary Fibre, 1990–2021.
